# *Pseudomonas aeruginosa* High-Risk Sequence Type 463 Co-Producing KPC-2 and AFM-1 Carbapenemases, China, 2020–2022

**DOI:** 10.3201/eid2910.230509

**Published:** 2023-10

**Authors:** Piaopiao Zhang, Wenhao Wu, Nanfei Wang, Haiting Feng, Jie Wang, Fang Wang, Yan Zhang, Hongchao Chen, Qing Yang, Yan Jiang, Tingting Qu

**Affiliations:** The First Affiliated Hospital of Zhejiang University School of Medicine, Hangzhou, China (P. Zhang, W. Wu, N. Wang, H. Feng, J. Wang, F. Wang, Y. Zhang, H. Chen, Q. Yang, T. Qu);; Zhejiang University School of Medicine Sir Run Run Shaw Hospital, Hangzhou (Y. Jiang);; Key Laboratory of Microbial Technology and Bioinformatics of Zhejiang Province, Hangzhou (Y. Jiang)

**Keywords:** *Pseudomonas aeruginosa*, ST463, sequence type, *bla*_KPC-2_, *bla*_AFM-1_, clonal spread, carbapenemase, KPC-2, AFM-1, antimicrobial resistance, bacteria, respiratory infections, China

## Abstract

We report the clonal spread and evolution of high-risk *Pseudomonas aeruginosa* sequence type 463 co-producing KPC-2 and AFM-1 carbapenemases isolated from hospital patients in China during 2020–2022. Those strains pose a substantial public health threat and surveillance and stricter infection-control measures are essential to prevent further infections.

Carbapenemase-producing *Pseudomonas aeruginosa* poses a global threat to public health. Epidemics caused by this pathogen are associated with high-risk clones (e.g., sequence type [ST] 235, ST277, ST175, ST233 and ST111), particularly those clones producing metallo-β-lactamases; Verona integron-encoded metallo-β-lactamase and imipenemase are the most prevalent carbapenemase types (*1*,*2*). A *Klebsiella* *pneumoniae* carbapenemase (KPC)–producing clone, ST463, has emerged and become predominant in carbapenemase-producing *P*. *aeruginosa* populations in China (*3*). Three *P. aeruginosa* strains co-producing KPC and *Alcaligenes* *faecalis* metallo-β-lactamase (AFM) were reported in 2022 and attributed to ST463 (*4*,*5*). During 2020–2022, we observed the clonal spread of ST463 carbapenem-resistant *P. aeruginosa* (CRPA) co-producing KPC-2 and AFM-1 (KPC-2–AFM-1 CRPA) in a hospital in China, which caused infections with high mortality. We report on KPC-2–AFM-1 CRPA emergence and driving forces that caused dissemination.

## The Study

During September 2020–June 2022, 192 nonduplicated CRPA isolates were collected from 192 patients admitted to a tertiary hospital in Zhejiang, China. Among those isolates, carbapenemase-producing *Pseudomonas aeruginosa* belonging to 10 different STs reached an overall prevalence of 41.1% (79/192) (Appendix Figure 1). We investigated KPC-2–AFM-1 CRPA strains isolated from 8 patients; 3 strains were colonizers and 5 were associated with infections (Table). The patients (6 men, 2 women) were 45–90 years of age, and all had complicated conditions and a history of intensive care unit admission. Antimicrobial drugs active against KPC-2–AFM-1 CRPA (colistin in monotherapy) were given to 4 patients; 5 infected patients eventually died; the remaining 3 patients, who only had bacterial colonization, were discharged (Table).

**Table:** Demographics and clinical characteristics of 8 patients infected by *Pseudomonas aeruginosa* high-risk sequence type 463 co-producing KPC-2 and AFM-1 carbapenemases, China, 2020–2022*

Patient no.	Strain	Age, y/sex	Ward	Underlying conditions	Sample isolation date	Sample type	Infection type	Therapy†	Outcome/Date
1	ZY94	90/M	ICU	Chronic kidney disease	2021 Feb 9	Sputum	Pulmonary infection	IMP, PTZ, COL	Death/2021 Feb 10
2	ZY156	77/M	ED	Acute toxic encephalopathy	2021 Feb 25	Urine	Urinary tract colonization	SCF	Discharge/2021 Mar 13
3	ZY1012	63/F	ICU	Intraabdominal malignancy	2021 Jul 26	Ascites	Intraabdominal infection	CZA, LEV, COL	Death/2021 Aug 20
4	ZY1075	79/M	ICU	Chronic cardiopulmonary disease	2021 Aug 12	Urine	Urinary tract colonization	NA	Discharge/2021 Aug 13
5	ZY1167	71/M	ICU	Hematologic malignancy	2021 Aug 28	Sputum	Pulmonary infection	IMP, PTZ, COL	Death/2021 Sep 13
6	ZY1214	45/M	Cardiac surgery	Aortic dissection	2021 Aug 31	Urine	Urinary tract colonization	SCF	Discharge/2021 Sep 8
7	ZY36	61/M	ICU	Chronic kidney disease	2022 Jan 9	Sputum	Pulmonary infection	IMP, SCF, COL	Death/2022 Jan 17
8	ZY1710	90/F	Geriatrics	Gallbladder carcinoma	2022 Apr 16	Sputum	Pulmonary infection	IMP	Death/2022 Apr 17

*AFM, *Alcaligenes* *faecalis* metallo-β-lactamase; COL, colistin; CZA, ceftazidime/avibactam; ED, emergency department; ICU, intensive care unit; IMP, imipenem; KPC, *Klebsiella* *pneumoniae* carbapenemase; LEV, levofloxacin, NA, not applicable (none); PTZ, piperacillin/tazobactam; SCF, cefperazone/sulbactam. †Therapy began after bacterial isolation and identification.

All 8 isolates were ST463, had identical resistance genes (Appendix Table 1) and type III secretion system genotype *exoU*+/*exoS*+, and differed by 5–30 single-nucleotide polymorphisms, indicating clonal dissemination (Appendix Figure 2). Each strain had 1 chromosome and a plasmid containing the β-lactamase gene *bla*_KPC-2_. Alignment of the 8 plasmids showed an identical backbone, but we noted major differences within the insertion region; the conserved core *bla*_KPC-2_ genetic platform insertion sequence (IS)*Kpn27*–*bla*_KPC-2_–IS*Kpn6* in plasmid p94 remained intact, whereas IS*Kpn6* and several genes immediately upstream (IS*Kpn6*–open reading frame–*klcA*–open reading frame) were absent in the other 7 plasmids (Appendix Figure 3, panel A), forming a novel genetic context for *bla*_KPC-2_. All plasmids were designated as type I plasmids (*6*) but had a 16-kb deletion of a mobilization-related operon within the backbone (Appendix Figure 3, panel B), supporting their nontransferability, which was confirmed by conjugation assays.

**Figure 1:**
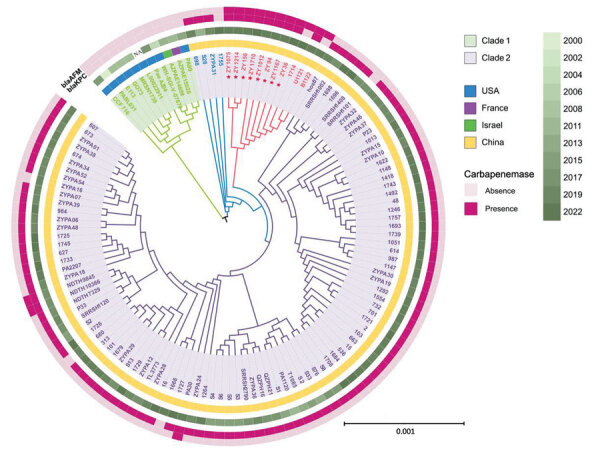
Phylogenetic analysis of *Pseudomonas aeruginosa* high-risk sequence type 463 strains co-producing KPC-2 and AFM-1 carbapenemases, China, 2020–2022. Core-genome phylogenetic tree was built for 125 *P. aeruginosa* sequence type 463 strains and rooted in the midpoint. Red asterisks indicate the 8 carbapenem-resistant *P. aeruginosa* strains co-producing KPC-2 and AFM-1 from a hospital in China. Presence and absence of *bla*_AFM_ and *bla*_KPC_ genes are indicated in the outermost 2 rings. Collection year, indicated by different shades of green, and location where each strain was isolated are indicated in the middle rings. The innermost shaded ring indicates strains belonging to clade 1 or 2. Scale bar indicates nucleotide substitutions per site. AFM, *Alcaligenes* *faecalis* metallo-β-lactamase; *bla*, β-lactamase; KPC, *Klebsiella* *pneumoniae* carbapenemase; NA, not available.

Using phylogenetic analysis of 125 ST463 genomes (68 from the National Center for Biotechnology Information Reference Sequence database [https://www.ncbi.nlm.nih.gov/refseq] and 57 from our collection), we found that those genomes were divided into 2 clades (Figure 1). Collection dates for strains in clade 1 were much earlier than those for clade 2. Clade 1 was primarily represented by clones from the United States, and clade 1 isolates did not harbor *bla*_KPC_ or *bla*_AFM_. The larger clade 2 originated in China and most isolates in this clade carried *bla*_KPC_; we also detected *bla*_AFM_ in clade 2. Independent evolution of ST463 clones in China might be correlated with sequential acquisition of *bla*_KPC_ and *bla*_AFM_. Paired single-nucleotide polymorphism distances for clade 2 strains in a minimum spanning tree were mainly 0–60 (Appendix Figure 2), demonstrating a high degree of relatedness among those isolates and clonal transmission of KPC-producing *P*. *aeruginosa* ST463 in China. The 8 KPC-2–AFM-1 CRPAs clustered with 3 *bla*_AFM_-carrying strains and formed a separate subclade inside clade 2 that was surrounded by *bla*_AFM_-negative, KPC-producing *P*. *aeruginosa*. Therefore, we inferred that ST463 KPC-2–AFM-1 CRPA clones probably arose from ST463 KPC-producing *P*. *aeruginosa*.

We could not map the genome sequences of clade 1 strains to the reference plasmid p94 (0% coverage), indicating the absence of type I plasmids in ST463 clones outside of China. Using BLAST (https://blast.ncbi.nlm.nih.gov), we retrieved 40 homologous plasmids that had >50% coverage and >95% identity to p94; all were type I plasmids carried by 14 different *P. aeruginosa* STs (Appendix Figure 4), suggesting an extensive horizontal transfer of type I plasmids within a narrow host range. Plasmid phylogeny revealed that all *bla*_KPC-2_–encoding plasmids clustered together in an independent branch, and separation of that branch from closely related *bla*_KPC-2_–negative plasmids might be associated with acquisition of *bla*_KPC-2_ (Appendix Figure 5). The presence of multiple copies of IS*26* in the *bla*_KPC-2_–adjacent region beyond the core platform indicated a critical role for IS*26* elements in remodeling and resistance evolution of type I plasmids in the ST463 lineage (Appendix Figure 4) (*7*). We deduced an underlying evolutionary pathway: an ST463 *P. aeruginosa* progenitor initially acquired a highly transferable type I plasmid, which subsequently evolved into a resistance plasmid through IS*26*-mediated insertion events involving a *bla*_KPC-2_–carrying region, then by chromosomal integration of *bla*_AFM-1_ to form KPC-2–AFM-1 CRPA. To further verify this hypothesis, we conducted a genomic comparison between ZY94 and the 1755 strain, which carried neither *bla*_KPC_ nor *bla*_AFM_ but was phylogenetically closest to the KPC-2–AFM-1 CRPA cluster (Figure 1). The genome of 1755 was also composed of 1 chromosome and 1 plasmid (p1755). Plasmid p94 was highly homologous (coverage 81%, identity 99.76%) to p1755 and might have evolved from p1755 via IS*26*-mediated intermolecular transposition of a *bla*_KPC-2_–carrying translocatable unit that targeted an existing copy of IS*26* (Appendix Figure 6, panel A). Comparisons between their chromosomes identified a redundant *bla*_AFM-1_–containing, multidrug-resistance fragment bracketed by IS*5564* in the same orientation, which was flanked by two 6-bp (GCTAGA) target site duplications (Appendix Figure 6, panel B), indicating a site-specific insertion event.

All 8 isolates were resistant to β-lactams and β-lactam/β-lactamase inhibitors, including carbapenems, ceftazidime/avibactam, fluoroquinolones, and gentamicin, and were only susceptible to amikacin and intermediately susceptible to colistin. We observed synergistic inhibitory effects against all 8 strains when we used combinations of ceftazidime/avibactam and aztreonam (Appendix Table 1). Compared with *P*. *aeruginosa* strain ATCC27853, all strains showed 4-fold higher MICs for chlorhexidine, a commonly used medical disinfectant, suggesting chlorhexidine tolerance (Appendix Table 1) (*8*). 

**Figure 2:**
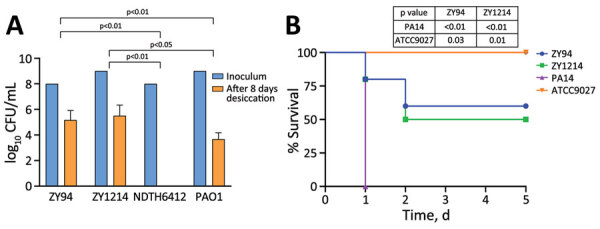
Desiccation tolerance and virulence analyses of *Pseudomonas aeruginosa* high-risk sequence type 463 co-producing KPC-2 and AFM-1 carbapenemases, China, 2020–2022. A) Carbapenem-resistant *P*. *aeruginosa* strains ZY94 and ZY1214 isolated from hospital patients in China were evaluated for desiccation tolerance. Results are given as mean (SD) CFU/mL for each isolate before and after 8 days of desiccation. Laboratory reference strain PAO1 and *P. aeruginosa* NDTH6412, which belongs to high-risk international sequence type 235, were used as controls. B) Virulence was determined by using Kaplan-Meier survival curves of mice intraperitoneally challenged with 1 × 10^7^ CFUs of strain ZY94 or ZY1214, hypervirulent strain PA14, or nonvirulent *P*. *aeruginosa* strain ATCC9027. Mice (10 per group) were monitored for 5 days and the number of dead mice was assessed each day. Mantel-Cox log rank tests were used to calculate p values for survival curve comparisons between the different strains. AFM, *Alcaligenes* *faecalis* metallo-β-lactamase; CFUs, colony forming units; KPC, *Klebsiella* *pneumoniae* carbapenemase.

All 8 strains exhibited small colony variant phenotypes together with strong biofilm formation capacities (Appendix Table 2). To assess their stability, desiccation resilience, and virulence, we selected strains ZY94 (infecting strain) and ZY1214 (colonizing strain) for further experiments. After we subcultured in antimicrobial drug–free Luria-Bertani broth for 10 days, ZY94 and ZY1214 retained stable small colony variant phenotypes; their *bla*_KPC-2_–carrying plasmid and *bla*_AFM-1_ gene were both stably inherited (stability was 93% for ZY94 and 95% for ZY1214). Both strains survived for 8 days on a dry polystyrene surface at higher rates than strains PAO1 and NDTH6412 (Figure 2, panel A). Furthermore, in mouse intraperitoneal challenge models (Appendix), ZY94 and ZY1214 strains had significantly higher (≈50%) lethality than ATCC9027 (p<0.05), but were less virulent than the PA14 strain (Figure 2, panel B). 

## Conclusions

We documented the persistent clonal spread of ST463 KPC-2–AFM-1 CRPAs in a hospital in China and provided insights into a potential evolutionary pathway for KPC-2–AFM-1 CRPA formation. Extensive drug resistance, disinfectant and desiccation resilience, strong biofilm formation, and high stability constituted strategies for those strains to defend against host and clinical challenges, thereby driving persistent transmission of this high-risk clone. Infections caused by such pathogens might lead to high death rates, especially in immunocompromised or critically ill patients, highlighting the urgent need for effective infection prevention and control policies. ST463 KPC-2–AFM-1 CRPA strains pose a substantial public health threat because of their extensive drug resistance, considerable pathogenicity, and ability to persist in the environment. Targeted surveillance and stricter infection-control measures are essential to prevent further infection outbreaks.

**Appendix.** Additional information for *Pseudomonas aeruginosa* high-risk sequence type 463 co-producing KPC-2 and AFM-1 carbapenemases, China, 2020–2022.
