# Supplementary material for: Pseudomonas aeruginosa High-Risk Sequence Type 463 Co-Producing KPC-2 and AFM-1 Carbapenemases, China, 2020–2022
Source: Emerg Infect Dis. 2023 Oct;29(10):2136–40. doi: 10.3201/eid2910.230509 (PMC10521588; doi:10.3201/eid2910.230509)
Supplement: Supplementary file 1 — Appendix. Additional information for Pseudomonas aeruginosa high-risk sequence type 463 co-producing KPC-2 and AFM-1 carbapenemases, China, 2020–2022. [file 23-0509-Techapp-s1.pdf]

Article DOI: https://doi.org/10.3201/eid2910.230509

EID cannot ensure accessibility for supplementary materials supplied by authors. Readers who have difficulty accessing supplementary content should contact the authors for assistance.

Pseudomonas aeruginosa High-Risk Sequence Type 463 Co-Producing KPC-2 and AFM-1 Carbapenemases, China, 2020–2022

Appendix

Detailed Methods

Bacterial Isolation and Clinical Data Collection

During September 2020–June 2022, a total of 192 nonduplicated clinical carbapenem-resistant *Pseudomonas aeruginosa* (CRPA) isolates were collected from patients in a tertiary hospital in Zhejiang, China, and were subjected to whole-genome sequencing for further molecular investigation. Eight CRPA strains co-producing *Klebsiella pneumoniae* carbapenemase (KPC) and *Alcaligenes faecalis* metallo-β-lactamase (AFM) (KPC-2–AFM-1 CRPA strains) were identified. Demographics and relevant clinical data were obtained through review of medical records. An infected case-patient was defined according to the US Centers for Disease Control and Prevention criteria (*1*); a colonized case-patient was defined as a patient carrying CRPA without clinical evidence of infection.

Antimicrobial Susceptibility Testing

MICs for 13 routine antimicrobial agents used to treat *P. aeruginosa* and chlorhexidine, a commonly used medical disinfectant, were determined by using the broth microdilution method. Clinical breakpoints of all antimicrobial drugs were interpreted according to the 2021 Clinical and Laboratory Standards Institute guidelines (*2*); *P. aeruginosa* ATCC27853 and *K. pneumoniae* ATCC700603 served as quality control strains. Accepted interpretive criteria have not been defined for disinfectants according to MIC values; therefore, comparing obtained results with those of the reference strain ATCC27853 might prove valuable.

Checkerboard Susceptibility Assay

In vitro synergistic inhibitory activities of ceftazidime/avibactam and aztreonam against all 8 isolates were determined in duplicate by using the broth microdilution checkerboard assay as previously described (*3*). The concentrations tested ranged from $\leq$1/32 × MIC to 2 × MIC for each antimicrobial drug. The interactions between antimicrobial agents were determined by calculating the fractional inhibitory concentration index (FICI). FICIs were interpreted as follows: FICI $\leq$0.5 = synergy, FICI >0.5 to $\leq$1 = additive, FICI >1 to $\leq$4 = no interaction, and FICI >4 = antagonism.

Whole-Genome Sequencing and Bioinformatics Analysis

Genomic DNA from 192 isolates was extracted and then sequenced by using the Illumina HiSeq X Ten platform (Illumina, https://www.illumina.com). Clean reads generated by Illumina sequencing were de novo assembled by using Shovill version 0.9.0 (https://github.com/tseemann/shovill). Long reads were sequenced for 8 KPC-2–AFM-1 CRPA isolates by using a MinION sequencer device (Oxford Nanopore Technologies, https:/www.nanoporetech.com). Hybrid assemblies of Illumina short reads and MinION long reads were prepared with Unicycler version 0.4.8 (*4*). Multilocus sequence typing was performed by using an mlst tool (https://github.com/tseemann/mlst). Antimicrobial drug resistance and virulence genes were identified with ABRicate version 1.0.0 (https://github.com/tseemann/abricate) according to the National Center for Biotechnology Information (NCBI) AMRFinderPlus (https://www.ncbi.nlm.nih.gov) and virulence factor (http://www.mgc.ac.cn/VFs) databases. Pairwise single-nucleotide polymorphism (SNP) distance was evaluated with Snp-dists (https://github.com/tseemann/snp-dists). Sequence comparisons were performed by using Easyfig version 2.2.3 and annotated by BacAnt and Prokka (*5*,*6*). Alignments and visualization of plasmids were generated by BLAST Ring Image Generator (BRIG) software version 0.95 (https://sourceforge.net/projects/brig).

Phylogenetic Analysis

A phylogenetic tree for all ST463 *P. aeruginosa* genomes from the NCBI database and our collection was constructed according to their SNPs by using Snippy version 4.4.5 (https://github.com/tseemann/snippy) and FastTree (*7*). The genome of strain B1122 (GenBank accession no. JAMWMN000000000) was used as a reference. As of September 2022, a total of 6,885 *P. aeruginosa* assembled genomes had been deposited in the NCBI Reference Sequence

database (https://www.ncbi.nlm.nih.gov/refseq), among which 68 genomes were assigned to ST463 and were downloaded for phylogenetic analysis.

The maximum likelihood phylogenetic tree for p94-related plasmid sequences was inferred from a core genome alignment by using Panaroo version 1.2.7 (*8*) and IQ-TREE version 2.1.2 (*9*). All trees were visualized in iTOL (https://itol.embl.de).

Plasmid Conjugation

The transferability of plasmids p94 and p1214 was evaluated by conjugation experiments; the filter mating method was used with a rifampin-resistant derivative of *P. aeruginosa*, PAO1, as the recipient strain. Transconjugants were selected on Mueller-Hinton agar plates supplemented with rifampin (800 μg/mL) and meropenem (4 μg/mL). Experiments were independently repeated 3 times.

Stability of bla_{KPC-2}–Carrying Plasmid and Chromosome-Encoded bla_{AFM-1}

To determine the stability of the bla_{KPC-2}–carrying plasmid and chromosomal bla_{AFM-1} gene, a passaging experiment was completed under antibiotic-free conditions. Briefly, 3 separate bacterial cultures were prepared by inoculating 3 mL Luria-Bertani (LB) broth without antibiotics and incubating overnight at 37°C, then a serial passage of 3 μL overnight culture into 3 mL LB was prepared each day. Samples were collected and diluted on Mueller-Hinton agar plates on day 10. Then, 48 colonies were randomly selected for validation of the presence of bla_{KPC-2}, *repA*, and bla_{AFM-1} by PCR with gene-specific primers. Retention was calculated as the percentage of cells with bla_{KPC-2}, *repA*, and bla_{AFM-1} in the 48 selected colonies.

Biofilm Quantification and Desiccation Tolerance

Biofilm quantification was performed by using crystal violet staining as described previously (*10*). Biofilm formation capabilities were assessed according to a published study (*11*). The capacity to survive on dry surfaces over time was evaluated as described previously with some minor modifications (*12*). Briefly, 100 μL of an overnight bacteria culture was serially diluted and counted the next day. Another 100 μL of the same culture was transferred into a 96-well microtiter plate; 3 technical replicates were prepared. The plate was air dried overnight and then incubated at 37°C for 8 days. Subsequently, 100 μL/well of fresh LB broth was added to the plate, and the plate was incubated with shaking at 37°C for 3 h. From each well, 100 μL of

suspension was collected, serially diluted, and counted the next day. Experiments were conducted in triplicate.

Mouse Peritonitis Model

Male immunocompetent BALB/c mice (6–8 weeks of age, weighing 20–25 g) were injected intraperitoneally with 100 μL phosphate-buffered saline solution containing 1×10^7 CFU of a *P. aeruginosa* strain in exponential growth phase (10 mice/group). PA14 was used as a hypervirulent reference strain, and *P. aeruginosa* ATCC9027 was a low-virulence reference strain (*13*). After inoculation, mice were monitored for 5 days, and the number of dead mice was assessed each day. All animal experiments were approved by the Institutional Animal Care and Ethics Committee of the First Affiliated Hospital of Zhejiang University School of Medicine.

Statistics

Statistical analysis was performed by using GraphPad Prism software, version 9.0 (GraphPad, https://www.graphpad.com). The Mann-Whitney U-test was applied for pairwise comparison of bacterial groups by using mean values. Survival curves for the mouse infection model were analyzed by log-rank (Mantel-Cox) test. p values of <0.05 were statistically significant.

Data Availability

The sequencing data for 8 isolates from this study have been deposited in GenBank under accession nos. JARDUM000000000, JARDUN000000000, JARDUO000000000, JARDUP000000000, CP125361-CP125362, CP125363-CP125364, CP125365-CP125366, and CP125367-CP125368.

References

1. Garner JS, Jarvis WR, Emori TG, Horan TC, Hughes JM. CDC definitions for nosocomial infections, 1988. Am J Infect Control. 1988;16:128–40. PubMed https://doi.org/10.1016/0196-6553(88)90053-3

2. Clinical and Laboratory Standards Institute. Performance standards for antimicrobial susceptibility testing; thirty-first edition (M100-Ed31). Wayne (PA): The Institute; 2021.

3. Al-Quraini M, Rizvi M, Al-Jabri Z, Sami H, Al-Muzahmi M, Al-Muharrmi Z, et al. Assessment of in-vitro synergy of fosfomycin with meropenem, amikacin and tigecycline in whole genome

sequenced extended and pan drug resistant *Klebsiella pneumoniae*: exploring a colistin sparing protocol. Antibiotics (Basel). 2022;11:153. PubMed https://doi.org/10.3390/antibiotics11020153

4. Wick RR, Judd LM, Gorrie CL, Holt KE. Unicycler: resolving bacterial genome assemblies from short and long sequencing reads. PLOS Comput Biol. 2017;13:e1005595. PubMed https://doi.org/10.1371/journal.pcbi.1005595

5. Hua X, Liang Q, Deng M, He J, Wang M, Hong W, et al. BacAnt: a combination annotation server for bacterial DNA sequences to identify antibiotic resistance genes, integrons, and transposable elements. Front Microbiol. 2021;12:649969. PubMed https://doi.org/10.3389/fmicb.2021.649969

6. Seemann T. Prokka: rapid prokaryotic genome annotation. Bioinformatics. 2014;30:2068–9. PubMed https://doi.org/10.1093/bioinformatics/btu153

7. Price MN, Dehal PS, Arkin AP. FastTree 2—approximately maximum-likelihood trees for large alignments. PLoS One. 2010;5:e9490. PubMed https://doi.org/10.1371/journal.pone.0009490

8. Tonkin-Hill G, MacAlasdair N, Ruis C, Weimann A, Horesh G, Lees JA, et al. Producing polished prokaryotic pangenomes with the Panaroo pipeline. Genome Biol. 2020;21:180. PubMed https://doi.org/10.1186/s13059-020-02090-4

9. Nguyen LT, Schmidt HA, von Haeseler A, Minh BQ. IQ-TREE: a fast and effective stochastic algorithm for estimating maximum-likelihood phylogenies. Mol Biol Evol. 2015;32:268–74. PubMed https://doi.org/10.1093/molbev/msu300

10. Zhang P, Wang J, Shi W, Wang N, Jiang Y, Chen H, et al. In vivo acquisition of bla_{KPC-2} with low biological cost in bla_{AFM-1}–harboring ST463 hypervirulent *Pseudomonas aeruginosa* from a patient with hematologic malignancy. J Glob Antimicrob Resist. 2022;31:189–95. PubMed https://doi.org/10.1016/j.jgar.2022.09.004

11. Macias-Valcayo A, Aguilera-Correa JJ, Broncano A, Parron R, Auñon A, Garcia-Cañete J, et al. Comparative in vitro study of biofilm formation and antimicrobial susceptibility in gram-negative bacilli isolated from prosthetic joint infections. Microbiol Spectr. 2022;10:e0085122. PubMed https://doi.org/10.1128/spectrum.00851-22

12. Heiden SE, Hübner NO, Bohnert JA, Heidecke CD, Kramer A, Balau V, et al. A *Klebsiella pneumoniae* ST307 outbreak clone from Germany demonstrates features of extensive drug resistance, hypermucoviscosity, and enhanced iron acquisition. Genome Med. 2020;12:113. PubMed https://doi.org/10.1186/s13073-020-00814-6

13. Grosso-Becerra MV, González-Valdez A, Granados-Martínez MJ, Morales E, Servín-González L, Méndez JL, et al. *Pseudomonas aeruginosa* ATCC 9027 is a non-virulent strain suitable for mono-rhamnolipids production. Appl Microbiol Biotechnol. 2016;100:9995–10004. PubMed https://doi.org/10.1007/s00253-016-7789-9

Appendix Table 1. Antimicrobial drug susceptibility profiles and resistance genes for the 8 ST463 KPC-2–AFM-1–CRPA isolates from China*

	MIC (µg/mL)														FICI		
Strain	MEM	IMP	CAZ	FEP	PIP	AZT	AK	GEN	LEV	CIP	P/T	CZA	COL	CHG	CZA/ AZT	Molecule	Antimicrobial resistance genes
ZY94	>128	>128	>128	>128	>128	>128	4	128	128	8	>128 /4	>128 /4	2	16	0.08	Chromosome	*aac(6')-Ib-G*, *aph(3″)-Ib*, *aph(3')-IIb*, *aph(6)-Id*, *bla*$_{\text{AFM-1}}$, *bla*$_{\text{CARB-2}}$, *bla*$_{\text{OXA-486}}$, *catB7*, *bla*$_{\text{PDC-374}}$, *ble*$_{\text{MBL}}$, *cmx* (×2), *crpP*, *floR2*, *fosA*, *mph(E)*, *msr(E)*, *sul1* (×2), *tet(G)*
																Plasmid	*bla*$_{\text{KPC-2}}$
ZY156	>128	>128	>128	>128	>128	>128	1	128	64	8	>128 /4	>128 /4	1	16	0.08	Chromosome	*aac(6')-Ib-G*, *aph(3″)-Ib*, *aph(3')-IIb*, *aph(6)-Id*, *bla*$_{\text{AFM-1}}$, *bla*$_{\text{CARB-2}}$, *bla*$_{\text{OXA-486}}$, *catB7*, *bla*$_{\text{PDC-374}}$, *ble*$_{\text{MBL}}$, *cmx* (×2), *crpP*, *floR2*, *fosA*, *mph(E)*, *msr(E)*, *sul1* (×2), *tet(G)*
																Plasmid	*bla*$_{\text{KPC-2}}$
ZY1012	>128	>128	>128	>128	>128	>128	4	128	64	16	>128 /4	>128 /4	2	16	0.08	Chromosome	*aac(6')-Ib-G*, *aph(3″)-Ib*, *aph(3')-IIb*, *aph(6)-Id*, *bla*$_{\text{AFM-1}}$, *bla*$_{\text{CARB-2}}$, *bla*$_{\text{OXA-486}}$, *catB7*, *bla*$_{\text{PDC-374}}$, *ble*$_{\text{MBL}}$, *cmx* (×2), *crpP*, *floR2*, *fosA*, *mph(E)*, *msr(E)*, *sul1* (×2), *tet(G)*
																Plasmid	*bla*$_{\text{KPC-2}}$
ZY1075	>128	>128	>128	>128	>128	>128	8	128	64	16	>128 /4	>128 /4	2	16	0.08	Chromosome	*aac(6')-Ib-G*, *aph(3″)-Ib*, *aph(3')-IIb*, *aph(6)-Id*, *bla*$_{\text{AFM-1}}$, *bla*$_{\text{CARB-2}}$, *bla*$_{\text{OXA-486}}$, *catB7*, *bla*$_{\text{PDC-374}}$, *ble*$_{\text{MBL}}$, *cmx* (×2), *crpP*, *floR2*, *fosA*, *mph(E)*, *msr(E)*, *sul1* (×2), *tet(G)*
																Plasmid	*bla*$_{\text{KPC-2}}$
ZY1167	>128	>128	>128	>128	>128	>128	4	128	64	16	>128 /4	>128 /4	1	16	0.16	Chromosome	*aac(6')-Ib-G*, *aph(3″)-Ib*, *aph(3')-IIb*, *aph(6)-Id*, *bla*$_{\text{AFM-1}}$, *bla*$_{\text{CARB-2}}$, *bla*$_{\text{OXA-486}}$, *catB7*, *bla*$_{\text{PDC-374}}$, *ble*$_{\text{MBL}}$, *cmx* (×2), *crpP*, *floR2*, *fosA*, *mph(E)*, *msr(E)*, *sul1* (×2), *tet(G)*
																Plasmid	*bla*$_{\text{KPC-2}}$
ZY1214	>128	>128	>128	>128	>128	>128	8	128	64	16	>128 /4	>128 /4	1	16	0.16	Chromosome	*aac(6')-Ib-G*, *aph(3″)-Ib*, *aph(3')-IIb*, *aph(6)-Id*, *bla*$_{\text{AFM-1}}$, *bla*$_{\text{CARB-2}}$, *bla*$_{\text{OXA-486}}$, *catB7*, *bla*$_{\text{PDC-374}}$, *ble*$_{\text{MBL}}$, *cmx* (×2), *crpP*, *floR2*, *fosA*, *mph(E)*, *msr(E)*, *sul1* (×2), *tet(G)*
																Plasmid	*bla*$_{\text{KPC-2}}$
ZY36	>128	>128	>128	>128	>128	>128	8	128	64	16	>128 /4	>128 /4	1	16	0.14	Chromosome	*aac(6')-Ib-G*, *aph(3″)-Ib*, *aph(3')-IIb*, *aph(6)-Id*, *bla*$_{\text{AFM-1}}$, *bla*$_{\text{CARB-2}}$, *bla*$_{\text{OXA-486}}$, *catB7*, *bla*$_{\text{PDC-374}}$, *ble*$_{\text{MBL}}$, *cmx* (×2), *crpP*, *floR2*, *fosA*, *mph(E)*, *msr(E)*, *sul1* (×2), *tet(G)*
																Plasmid	*bla*$_{\text{KPC-2}}$
ZY1710	>128	>128	>128	>128	>128	>128	8	128	64	16	>128 /4	>128 /4	1	16	0.27	Chromosome	*aac(6')-Ib-G*, *aph(3″)-Ib*, *aph(3')-IIb*, *aph(6)-Id*, *bla*$_{\text{AFM-1}}$, *bla*$_{\text{CARB-2}}$, *bla*$_{\text{OXA-486}}$, *catB7*, *bla*$_{\text{PDC-374}}$, *ble*$_{\text{MBL}}$, *cmx* (×2), *crpP*, *floR2*, *fosA*, *mph(E)*, *msr(E)*, *sul1* (×2), *tet(G)*
																Plasmid	*bla*$_{\text{KPC-2}}$
ATCC 27853	0.5	2	2	1	2	4	2	1	1	0.5	4/4	NA	0.5	4	NA	NA	NA

*AFM, *Alcaligenes faecalis* metallo-β-lactamase; AK, amikacin; ATCC, American Type Culture Collection (https://www.atcc.org); AZT, aztreonam; CAZ, ceftazidime; CHG, chlorhexidine; CIP, ciprofloxacin; COL, colistin; CRPA, carbapenem-resistant *Pseudomonas aeruginosa*; CZA, ceftazidime/avibactam; FEP, cefepime; FICI, fractional inhibitory concentration index; GEN, gentamicin; IPM, imipenem; KPC, *Klebsiella pneumoniae* carbapenemase; LEV, levofloxacin; MEM, meropenem; NA, not applicable; PIP, piperacillin; P/T, piperacillin/tazobactam; ST463, sequence type 463.

Appendix Table 2. Quantitative data for biofilm formation by 8 KPC-2–AFM-1 CRPA isolates from China*

Strain	Optical density at 590 nm								
ZY36	1.128	0.835	1.445	1.142	1.129	1.416	1.13	1.379	1.575
ZY94	1.039	0.897	0.921	0.73	1.023	1.072	0.811	0.866	1.402
ZY156	0.972	0.816	0.771	0.867	0.83	0.811	0.865	0.834	1.293
ZY1012	0.782	0.782	0.855	0.85	0.892	0.81	0.986	0.896	1.031
ZY1075	1.029	0.854	1.167	1.052	1.051	1.077	1.098	0.889	1.557
ZY1167	0.981	1.14	1.329	0.954	1.117	1.259	1.203	1.004	1
ZY1214	0.877	0.951	0.947	0.879	0.829	0.895	0.829	0.937	1.116
ZY1710	1.012	0.958	0.988	0.803	0.814	0.939	0.933	0.93	1.176
Negative control	0.113	0.119	0.133	0.111	0.144	0.115	0.124	0.123	0.113

*AFM, *Alcaligenes faecalis* metallo-β-lactamase; CRPA, carbapenem-resistant *Pseudomonas aeruginosa*; KPC, *Klebsiella pneumoniae* carbapenemase.

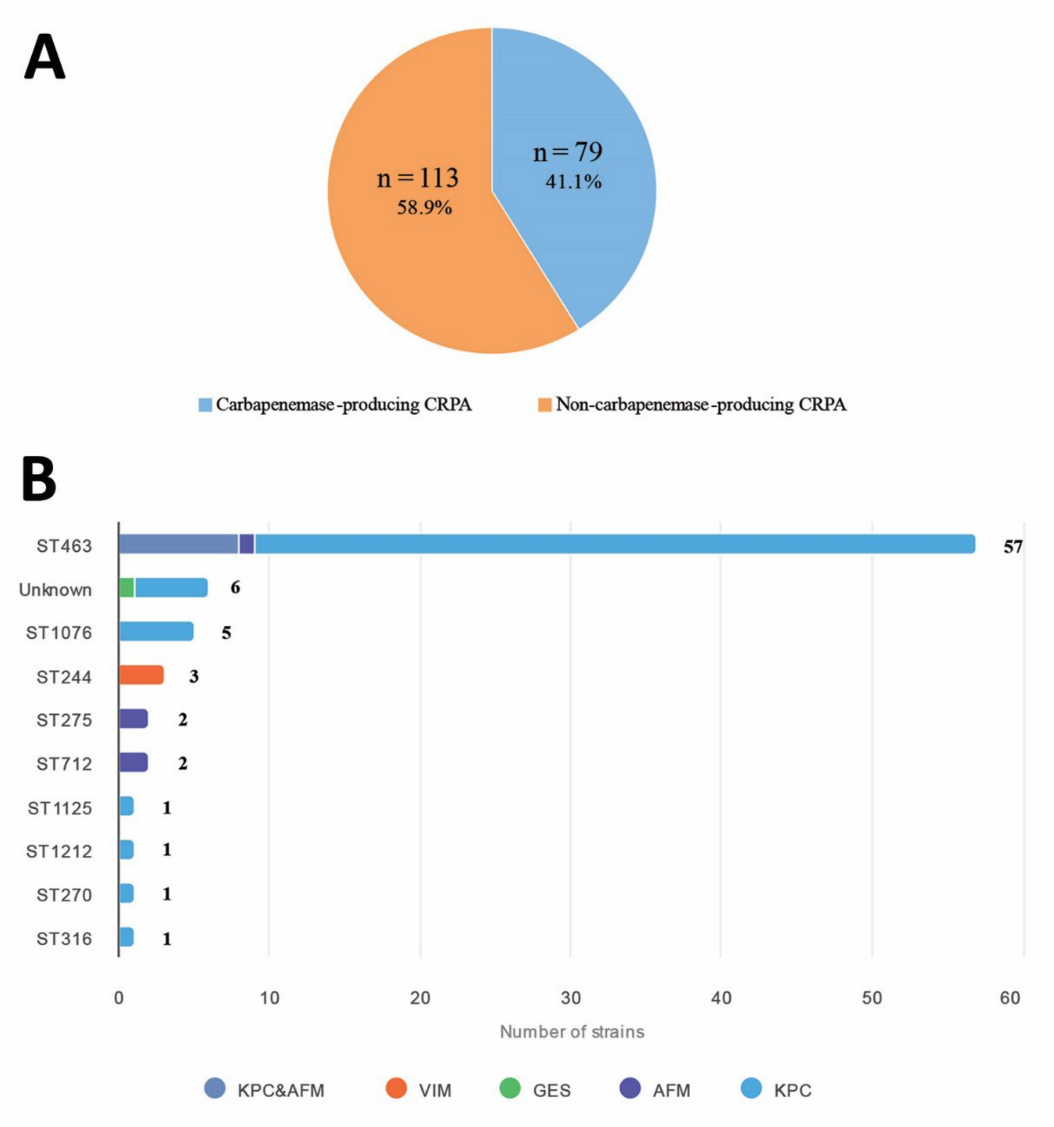

Appendix Figure 1. Distribution of carbapenem-resistant *Pseudomonas aeruginosa* strains isolated from hospital patients in Zhejiang, China during 2020–2022. A) Prevalence of carbapenemase-producing and noncarbapenemase-producing strains. B) Number and distribution of carbapenemases in 10

carbapenemase-producing *P. aeruginosa* sequence types. AFM, *Alcaligenes faecalis* metallo-β-lactamase; CRPA, carbapenem-resistant *P. aeruginosa*; GES, Guiana extended-spectrum β-lactamase; KPC, *Klebsiella pneumoniae* carbapenemase; ST, sequence type; VIM, Verona integron-encoded metallo-β-lactamase.

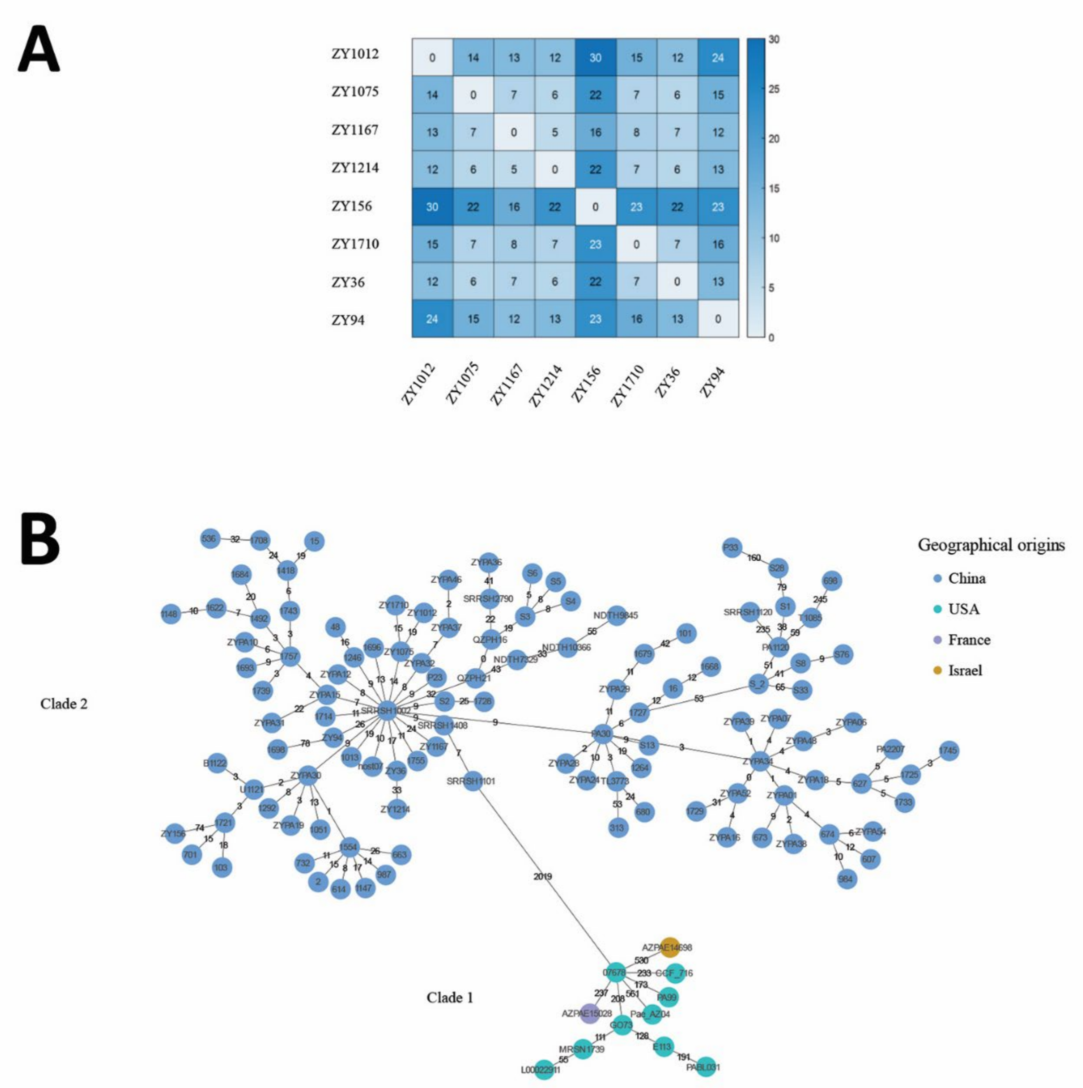

Appendix Figure 2. Single-nucleotide polymorphism distances and minimum spanning tree analysis of sequence type 463 carbapenem-resistant *Pseudomonas aeruginosa* strains isolated during this study that co-produced *Klebsiella pneumoniae* carbapenemase-2 and *Alcaligenes faecalis* metallo-β-lactamase-1. A) Paired single-nucleotide polymorphism (SNP) distances between each strain. B) Minimum spanning tree of 125 sequence type 463 isolates according to core SNPs. Each circle indicates an individual strain; different fill colors indicate geographic origin of each strain. Numbers on connecting lines indicate the number of SNPs in a pairwise comparison.

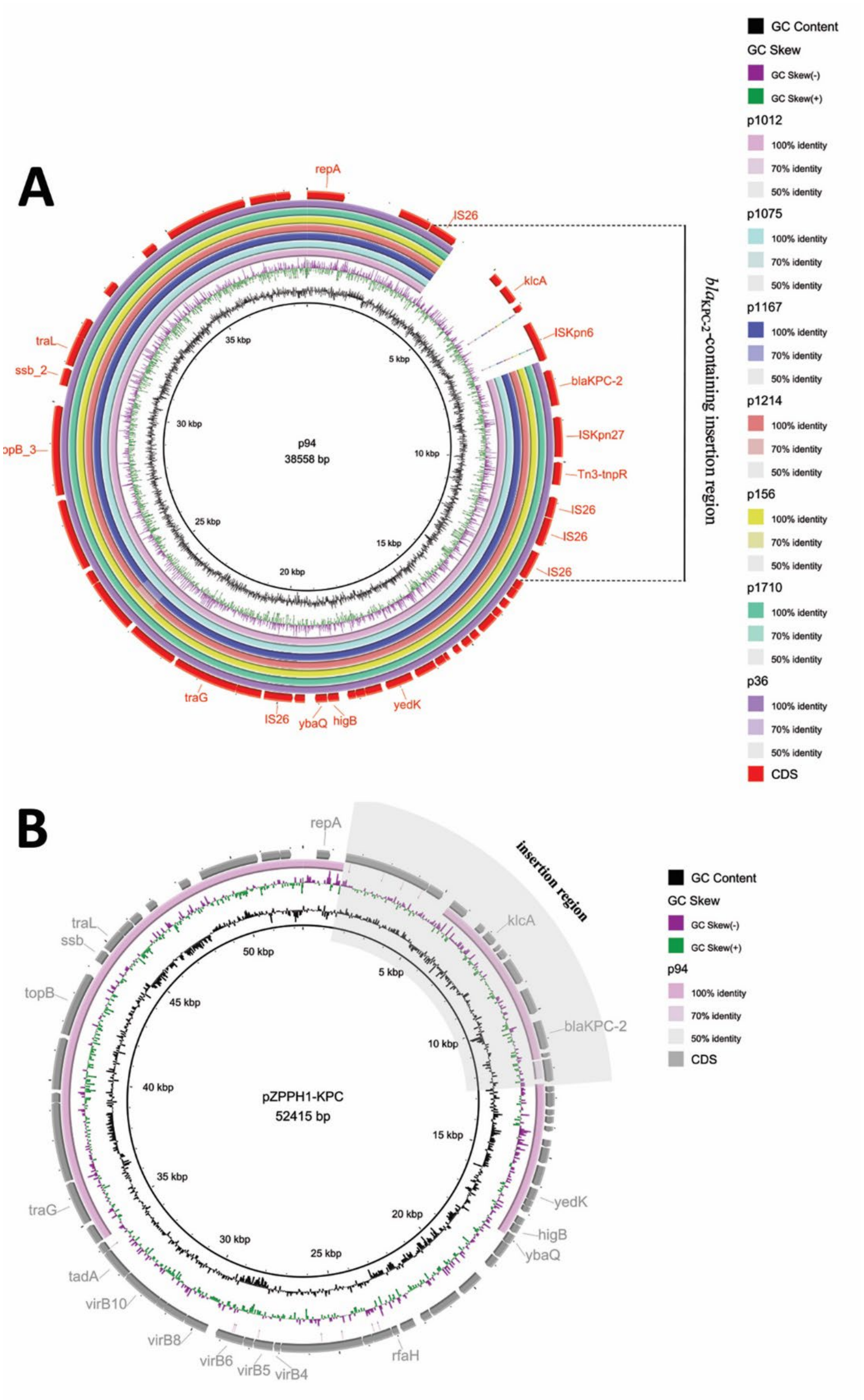

Appendix Figure 3. Alignments and genetic organization of plasmids from sequence type 463 carbapenem-resistant *Pseudomonas aeruginosa* strains isolated during this study that co-produced *Klebsiella pneumoniae* carbapenemase-2 and *Alcaligenes faecalis* metallo-β-lactamase-1. A) Alignment

of complete bla_{KPC-2}-containing plasmids from 8 carbapenem-resistant *P. aeruginosa* isolates obtained in this study. Plasmid p94 from strain ZY94 is the reference sequence. B) Genetic organization of archetypal type I plasmid pZPPH1-KPC (GenBank accession no. CP077990) and comparison with p94 isolated in this study. Structure of pZPPH1-KPC consists of backbone and insertion regions (shaded gray). Outermost ring is an annotation of the reference plasmids (p94 and pZPPH1-KPC) and shows the direction of the transcriptional open reading frame. GC-rich regions (GC skew) are indicated. CDS, coding sequence.

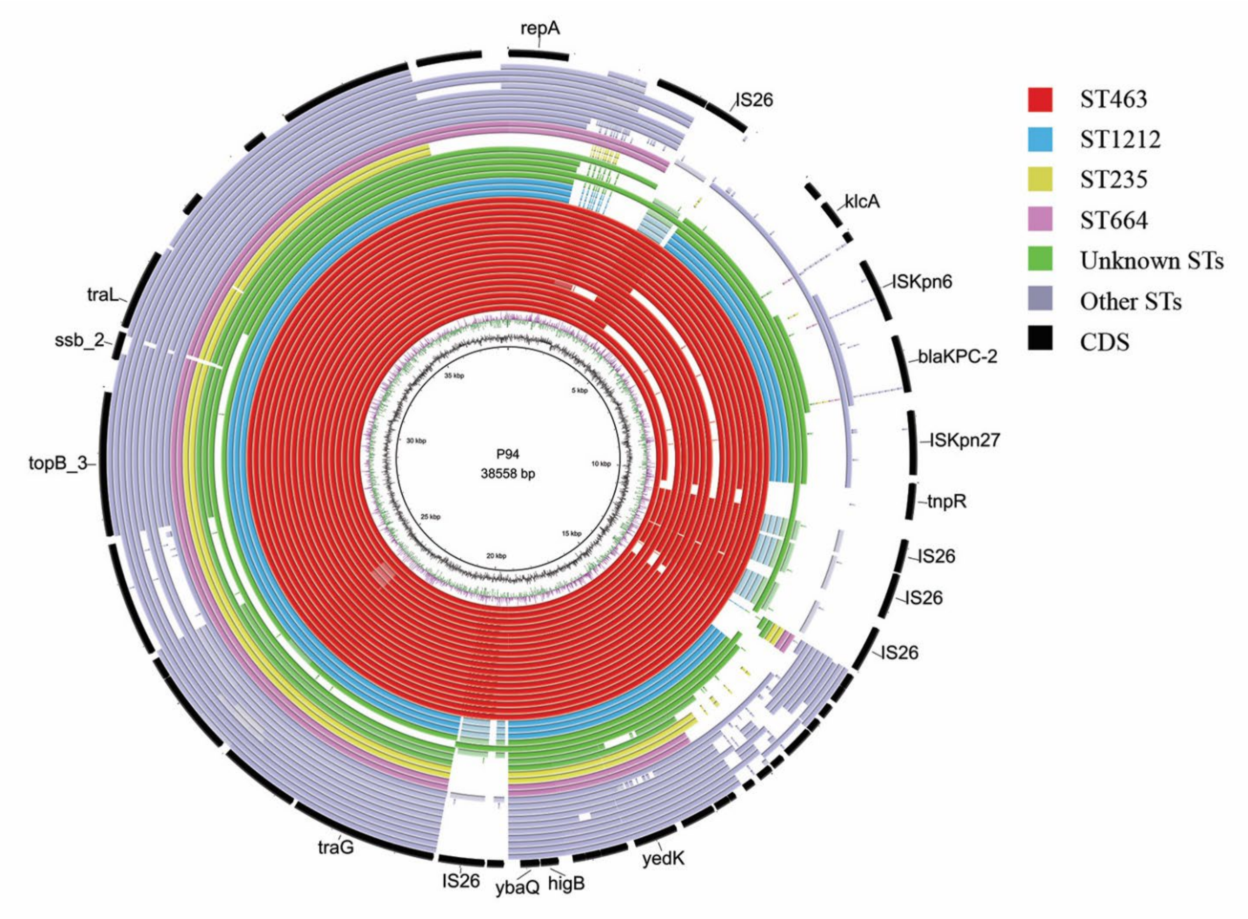

Appendix Figure 4. Comparison of plasmid p94 from this study and homologous plasmids isolated from other *Pseudomonas aeruginosa* strains. BLAST Ring Image Generator (https://sourceforge.net/projects/brig) diagram shows p94 in the innermost ring and alignments with 40 related plasmids. Colored rings indicate different *P. aeruginosa* sequence types. The outermost black ring shows genes on p94. CDS, coding sequence; ST, sequence type.

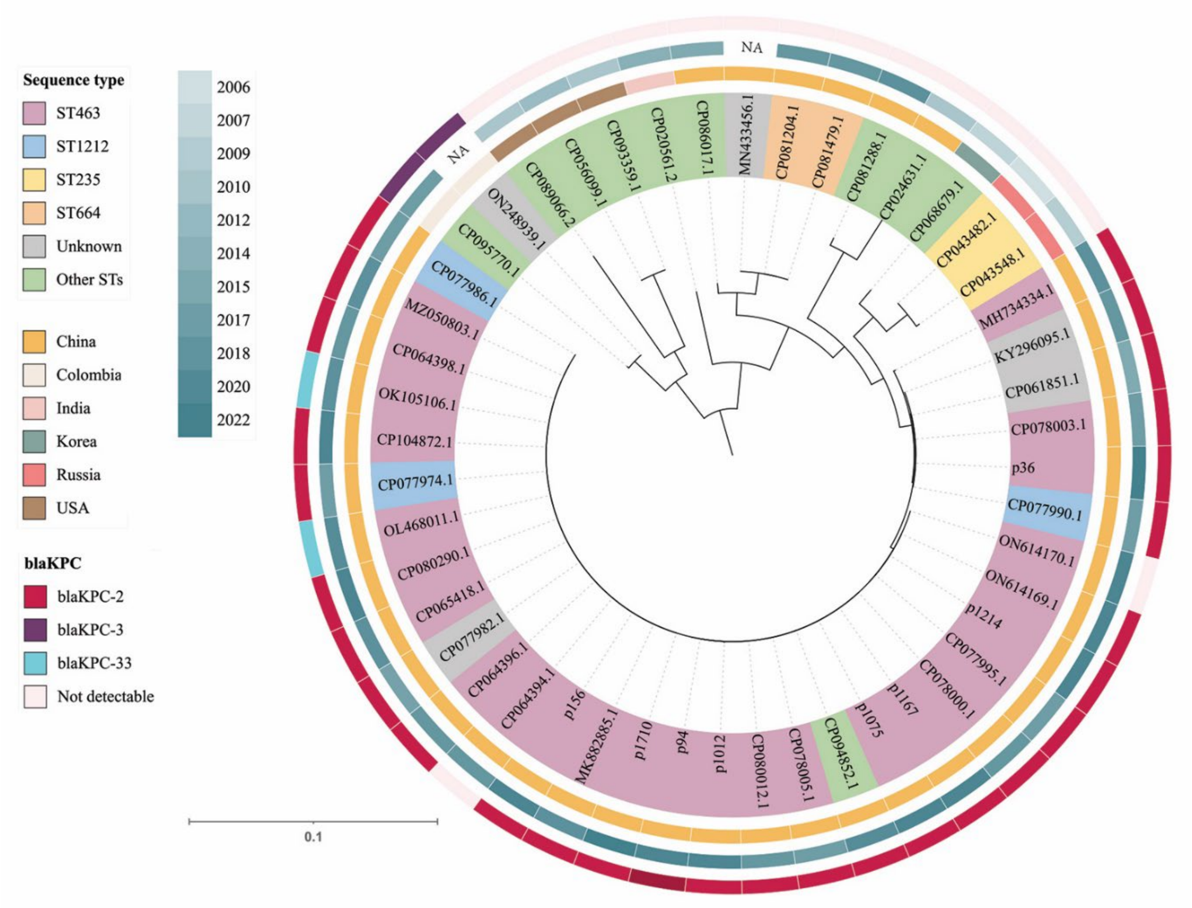

Appendix Figure 5. Phylogenetic analysis of 8 p94-related plasmids from this study and 40 related plasmids from GenBank. The outermost ring indicates the presence of different *Klebsiella pneumoniae* carbapenemase genes. The 2 middle rings indicate the isolate collection year and origin. GenBank accession nos. are indicated. Scale bar indicates nucleotide substitutions per site. NA, not available; ST, sequence type.

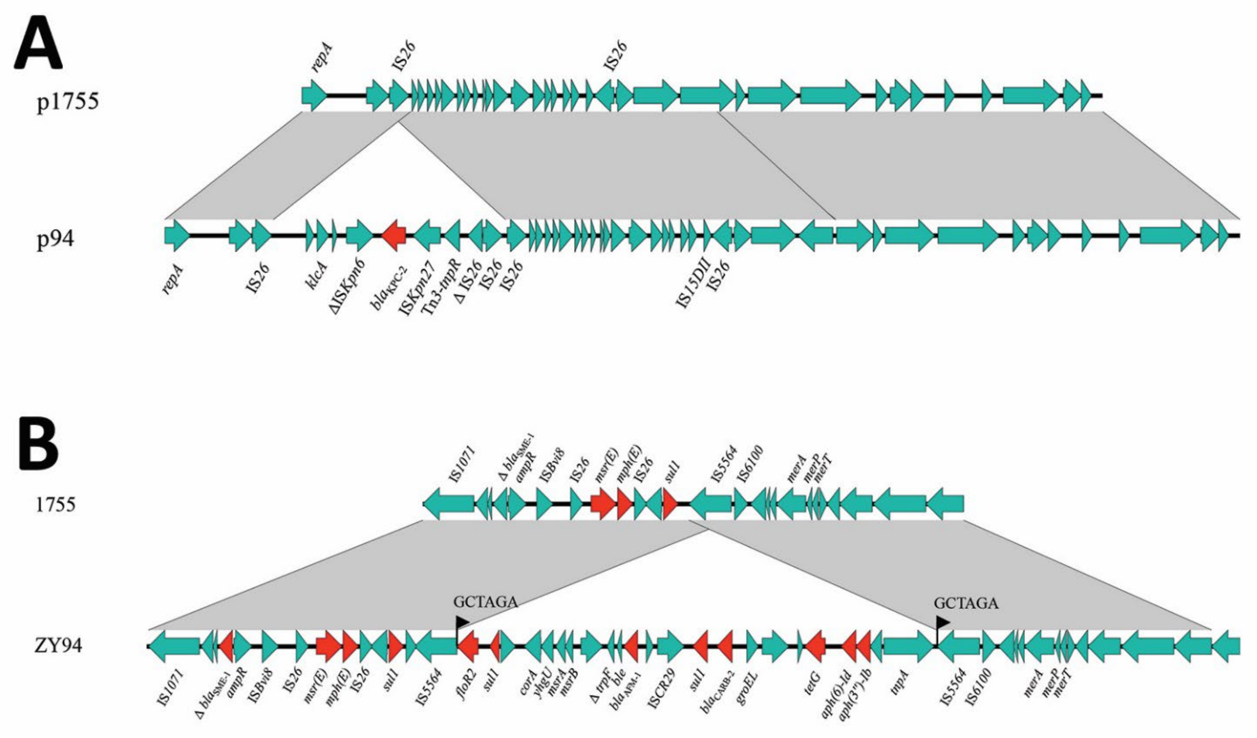

Appendix Figure 6. Genomic comparison of plasmid and chromosome sequences between strains ZY94 from this study and 1755. A) Co-linear genome alignment of plasmids p1755 and p94. B) Comparison of partial chromosomes of 1755 and ZY94. Colored arrows indicate open reading frames; resistance genes are marked with red triangles. Regions of >99.0% nucleotide sequence identity are shaded in gray. Black flags highlight the positions of target site duplications.
